# A SGLT2 inhibitor dapagliflozin suppresses prolonged ventricular-repolarization through augmentation of mitochondrial function in insulin-resistant metabolic syndrome rats

**DOI:** 10.1186/s12933-018-0790-0

**Published:** 2018-11-17

**Authors:** Aysegul Durak, Yusuf Olgar, Sinan Degirmenci, Erman Akkus, Erkan Tuncay, Belma Turan

**Affiliations:** 1 Departments of Biophysics, Faculty of Medicine, Ankara University, Ankara, Turkey; 2 Internal Medicine, Faculty of Medicine, Ankara University, Ankara, Turkey

**Keywords:** Diabetes, SGLT2 inhibitors, Heart function, Electrophysiology, Oxidative stress, Insulin resistance

## Abstract

**Background:**

Metabolic syndrome (MetS) is a prevalent risk factor for cardiac dysfunction. Although SGLT2-inhibitors have important cardioprotective effects in hyperglycemia, their underlying mechanisms are complex and not completely understood. Therefore, we examined mechanisms of a SGLT2-inhibitor dapagliflozin (DAPA)-related cardioprotection in overweight insulin-resistant MetS-rats comparison with insulin (INSU), behind its glucose-lowering effect.

**Methods:**

A 28-week high-carbohydrate diet-induced MetS-rats received DAPA (5 mg/kg), INSU (0.15 mg/kg) or vehicle for 2 weeks. To validate MetS-induction, we monitored all animals weekly by measuring body weight, blood glucose and HOMO-IR index, electrocardiograms, heart rate, systolic and diastolic pressures.

**Results:**

DAPA-treatment of MetS-rats significantly augmented the increased blood pressure, prolonged Q–R interval, and low heart rate with depressed left ventricular function and relaxation of the aorta. Prolonged-action potentials were preserved with DAPA-treatment, more prominently than INSU-treatment, at most, through the augmentation in depressed voltage-gated K^+^-channel currents. DAPA, more prominently than INSU-treatment, preserved the depolarized mitochondrial membrane potential, and altered mitochondrial protein levels such as Mfn-1, Mfn-2, and Fis-1 as well as provided significant augmentation in cytosolic Ca^2+^-homeostasis. Furthermore, DAPA also induced significant augmentation in voltage-gated Na^+^-currents and intracellular pH, and the cellular levels of increased oxidative stress, protein-thiol oxidation and ADP/ATP ratio in cardiomyocytes from MetS rats. Moreover, DAPA-treatment normalized the increases in the mRNA level of SGLT2 in MetS-rat heart.

**Conclusions:**

Overall, our data provided a new insight into DAPA-associated cardioprotection in MetS rats, including suppression of prolonged ventricular-repolarization through augmentation of mitochondrial function and oxidative stress followed by improvement of fusion–fission proteins, out of its glucose-lowering effect.

**Electronic supplementary material:**

The online version of this article (10.1186/s12933-018-0790-0) contains supplementary material, which is available to authorized users.

## Introduction

Metabolic syndrome (MetS) is a cluster of metabolic disorders and closely linked to overweight or obesity and inactivity. It is comprised of a combination of risk factors for coronary heart disease, as well as diabetes. There is an increasing evidence that a pathophysiological basis of MetS is various and complex, yet the microvascular function is a potential factor to explain a cluster of several components of MetS including hypertension, obesity/overweight-body and insulin resistance [1]. Even early studies have shown that there is a close association between obesity, hemodynamic overload [2], and atrial and ventricular remodeling [3, 4] which are exacerbated by concomitant hypertension [5]. MetS people are strongly advised to reduce their risk of cardiovascular disease and type 2 diabetes (T2DM) by control of risk factors.

Interestingly, epidemiological studies reported that there was an association between higher mortality rates and uncontrolled glycemic control, primarily through the use of insulin, in patients with both T2DM and heart failure [6, 7] via flooding the heart with energy-providing substrates, including fats and sugars with insulin and insulin-sensitizing medications [8]. However, some studies reported the possible effects of multiple insulin- or noninsulin-therapies on glycemic control and heart failure [8]. Nevertheless, few studies reported the action of sodium-glucose cotransporter-2 (SGLT2) inhibitors, which affected both supply and demand pathways in diabetics with chronic heart failure.

In this concept, Han et al. [9] described in vitro and in vivo pharmacological effects of the SGLT2-selective inhibitor dapagliflozin (DAPA), currently under a clinical investigation for use as an antidiabetic agent confirmed by studies with acute and multi-dose in normal and diabetic rats. They demonstrated that DAPA could improve fed and fasting plasma glucose levels and glucose utilization after multi-dose treatment with a conclusion on the potential role of DAPA as an efficacious treatment for T2DM. Zinman et al. performed a clinical study by use of a SGLT2 inhibitor, in addition to standard care, on cardiovascular morbidity and mortality in patients with T2DM at high cardiovascular risk (EMPA-REG OUTCOME Trial; median observation time, 3.1 years). They demonstrated that these patients had a lower rate of the primary composite cardiovascular outcome [10]. Since the report of this trial announced, these inhibitors were started to use primarily by clinicians for in favor of patients to prevent a cardiovascular disease, rather than focusing on glycemic control per se. However, the mechanisms of that SGLT2 inhibition improves cardiovascular outcomes are not fully understood.

Some human studies suggested an association between insulin resistance and abnormal mitochondrial activity in muscles [11, 12] while some others pointed out how a deficient mitochondrial function could converge to insulin resistance [13, 14]. In support of these previous studies, Sebastián et al. [15] demonstrated an important description of a unique coordinating role of mitofusion proteins in mitochondria and endoplasmic reticulum function, led to modulation of insulin signaling and glucose homeostasis liver-specific Mfn-2 KO-mice. On the basis of these studies, here, we aimed to examine the molecular mechanism of the beneficial effect of an SGLT2 inhibitor on cardiac dysfunction in insulin-resistant overweight MetS rats. For this purpose, we first examined the effect of DAPA-treatment on the function of the cardiovascular system in the MetS rats in both tissue and isolated cardiomyocyte levels. Moreover, we also aimed to determine in vivo effects of DAPA on a mitochondrial function by determination of mitochondrial membrane potential, mitofusion-fission proteins, and glucose homeostasis together with any augmentation of cellular oxidative stress under hyperglycemia and insulin resistance in MetS rats. Second, in order to demonstrate the cardioprotective action of DAPA through insulin-independent pathways, we compared all data with insulin-treated MetS rats. Our study provided a mechanism for a complete cardioprotective action of DAPA in vivo in overweight insulin-resistant MetS-rats through not an only augmentation of voltage-gated K^+^-channel currents but also mitochondrial function and increased oxidative stress, in part, under the control of fusion–fission proteins at the cellular level via insulin-independent pathways.

## Methods

### Animal model and treatment

Male Wistar rats with age of 2-month old were used for induction of metabolic syndrome (MetS) and the similar animal model was used as described, previously [16]. Briefly, MetS rats, following received 32% sucrose into their drinking water for 30 weeks (validated in this group by measuring body weight, fasting blood glucose level, insulin level, oral glucose tolerance test and the development of insulin resistance with this diet was determined by the HOMA-IR index), as described previously [16]. were treated either with dapagliflozin (DAPA; 5 mg/kg, Bristo-Myers Squibb Manufacturing Company, Humacao, Porto Riko), insulin (INSU; 0.15 mg/kg, Humalog Mix25 Kwikpen, Lilly) or vehicle (MetS) for 2 weeks. All animals are exposed to a 12-h light–dark cycle and the control group (CON) had also free access to tap water. All animals were fed standard chow ad libitum and are housed in the standard rat cages.

Systolic and diastolic blood pressures are measured by an indirect tail-cuff method via a NIBP200-A noninvasive blood pressure meter (BIOPAC Systems Inc, USA) as described previously [17].

### Electrocardiography recording

After completion of 2-week drug administrations, in situ ECGs were recorded through the animal’s paws using two custom-made electrodes (MP150, BIOPAC Systems, Inc.) under light ether anesthesia [16]. The ECGs are band-pass filtered (50–500 Hz). All animals had their ECG’s recorded at least for 10 min. The durations of ECGs such as PR-, RR- and QT-intervals, and heart rate were calculated.

### QRT-PCR analysis

Total-RNA was prepared using RNA Isolation-kit (Macherey–Nagel, 740955.10) from heart and kidney tissues, as described previously [18]. The purified total-RNA was reverse transcribed with ProtoScript First-Strand cDNA Synthesis-kit (New England Biolabs, E6300S). The amplified fragment size of PCR-products for each primer and primers’ specificity were controlled with NCBI and ENSEMBL databases. Primer sequence for SGLT2 and the validation of SGLT2 in the heart tissue were given in Additional file 1: Figure S1. The fold changes in the genes were analyzed based on comparative (2^−ΔΔCt^) method.

### Determination of left ventricular function with Langendorff-perfused heart

The left ventricular developed pressure (LVDP) and rates of pressure development and decay (± dP/dt) were measured in isolated hearts as described elsewhere [19]. Briefly, the hearts were pressure changes were electrically stimulated (DCS; Harward) at 300 beats/min with 1.5 ms square waves (at twice the threshold voltage) and pressure changes were measured with a water-filled latex balloon inserted into the left ventricle. All data were recorded online then stored and processed (Model 1050BP; BIOPAC Systems, Goleta, California, USA). The results are given as percentage changes with respect to the controls.

### Fresh cardiomyocyte isolation

Cardiomyocyte isolation was performed freshly from the left ventricle of the heart by an enzymatic method, as described previously [20]. Briefly, the heart was first perfused at 37 °C with a Ca^2+^-free, HEPES-buffered solution for 5 min. After that procedure, fresh buffer supplemented with 1.3 mg/ml collagenase (Type A, Boehringer) was perfused (6–8 ml/min) for 30 min. Then, the hearts were removed from the perfusion system and the left ventricle was cut off and stirred to disperse the cardiomyocytes. The cells were then suspended in HEPES buffer with 1 mM Ca^2+^ and 0.5% bovine serum albumin (pH 7.4). Cardiomyocytes were kept at 37 °C during the day (up to 6 h) for experiments.

### Electrophysiological measurements in isolated cardiomyocytes

All electrophysiological measurements in isolated cardiomyocytes were performed by using Axopatch 200B amplifier with the software of pCLAMP 10.0 Axon Instruments and Digidata 1440A analog-to-digital converter. The cell capacitance of cardiomyocytes was measured to determine cell size and all traces were sampled and digitized at 5 kHz and filtered at 3 kHz with the Digidata. Liquid-junction-potential was compensated before establishing the gigaseal and no leak or capacitance subtractions were performed in our current and voltage recordings. All records were measured with borosilicate glass capillary tubes with 2–3 MΩ to avoid internal dialysis.

*Action potentials* in left ventricular cardiomyocytes were determined under electrical stimulation at 0.5 Hz frequency, as described previously [21]. The pipette solution for action potential recording contained (in mmol/l); KCl 140, HEPES 25, Mg-ATP 3, EGTA 5, Na-GTP 0.4 at pH 7.2 with KOH. The parameters of action potentials such as the resting membrane potentials, the maximum depolarization potentials and the durations from repolarization phase at 25, 50, 75, 90% (APD_25, 50, 75, 90_) were calculated from original records.

*Whole*-*cell TTX*-*sensitive voltage*-*dependent Na*^+^-*channel currents (I*_*Na*_*)* in cardiomyocytes were recorded as described, previously [22]. Briefly, using a pre-pulse protocol (holding potential: − 80 to − 120 mV, followed by 200 ms depolarizing 5 mV voltage steps from − 70 to + 40 mV), the I_Na_ were recorded and calculated as a difference between negative peak and the current obtained at the end of the pulse. All current recordings were performed at room temperature and cells were superfused with a low Na^+^-HEPES solution of the following composition (mmol/l: NaCl 40, *N*-methyl-d-glucamine 77, CsCl 20, CaCl_2_ 1.8, MgCl_2_ 1.8, CdCl_2_ 0.2, glucose 10, HEPES 10 and pH at 7.4 with HCl). The internal solution of the patch pipette contained (in mmol/l); CsCl 120, Mg-ATP 5, HEPES 20, EGTA 5, Na-GTP 0.4, pH at 7.2 with CsOH.

*Whole*-*cell voltage*-*dependent the L*-*type Ca*^*2*+^-*currents (I*_*CaL*_*)* were recorded and calculated as described, previously [20]. The composition of the electrode solution was the following (in mmol/l): l-aspartic acid 120, CsCl 10, NaCl 10, HEPES 10, Mg-ATP 5, EGTA 10; pH adjusted to 7.2 with CsOH. Modified Tyrode solution containing (in mmol/l) NaCl 117, CsCl 20, MgCl_2_ 1.7, CaCl_2_ 1.8, HEPES 10 and glucose 10 was used for performing external perfusion. Voltage clamp protocol contained a pre-pulse from − 70 to − 55 mV (for inactivating the Na^+^ currents), followed by 300-ms depolarizing voltage steps between − 60 and + 80 mV.

*Whole*-*cell voltage*-*dependent K*^+^-*channel currents (I*_*K*_*)* were recorded as described, previously. The transient outward K^+^-current (I_to_) calculated as the difference between peak and the last part of the recorded K^+^-currents while inward rectifier K^+^-currents (I_K1_) were determined as the mean of the last 200-ms part of the signal.

For comparison, all recorded currents were divided by the cell membrane capacitance to present them as current density (in pA/pF).

### Determination of intracellular transient Ca^2+^ changes

Isolated cardiomyocytes, following loading with the fluorescent Ca^2+^ indicators Fura-2AM (4-µM), intracellular transient global free Ca^2+^ changes under electric-field stimulation (Ca^2+^-transients) were measured at room temperature (21 ± 2 °C), as described, previously [20]. Fluorescence was recorded using microspectrofluorometer and FELIX software (Photon Technology International, Inc., NJ USA) with excitation at 340 nm/380 nm and emission at 510 nm under electric-field stimulation with electrical pulses with 10-ms duration, at a frequency of 0.2 Hz. The fluorescence ratio (the difference between basal and peak F_340/380_) was used as an indicator of intracellular free Ca^2+^ changes in each cell.

### Determination of intracellular free ion levels in cardiomyocytes

To determine the basal levels of some intracellular free ions such as Ca^2+^, Na^+^, H^+^, or pH_i_, cardiomyocytes in resting state are first loaded with ion-specific fluorescence dye (Fura-2AM, SBFI, SNARF-1, respectively). Then, fluorescence recordings were by use of either a PTI Ratiomaster microspectrofluorometer and FELIX software (Photon Technology International) for Ca^2+^ and Na^+^ or a laser scanning microscope (confocal microscopy, Leica TCS SP5, Germany) for pH_i_, as described previously [22]. The fluorescence changes for Ca^2+^ and Na^+^, the loaded cells were excited at 340/380 nm and were obtained excitation at 340/380 nm and 350/380 nm, respectively while the emission was at 510 nm. Carboxy-SNARF-1 fluorescence from individual cells was excited at 543 nm of laser line of a helium–neon laser and measured simultaneously at 580 and 640 nm. Data were obtained in a rate of 1.5 Hz. The emission ratios referred as pH_i_ values. Background fluorescence measured from a cell-free field was subtracted from all recordings before the calculation of ratios.

### Determination of intracellular ROS and RNS levels in living cardiomyocytes using fluorescent probes

Measurement of ROS production was performed in CM-H2DCFDA loaded (5 µM with 45-min incubation at 37 °C) cardiomyocytes, as described previously [23]. Briefly, the fluorescence changes were recorded in the cells due to their response rate to H_2_O_2_ exposure (100 µM; maximum fluorescence intensity, F_max_) by using confocal microscopy (Leica TCS SP5, Germany). Immediately after addition, it was started to measuring the fluorescence in kinetic mode at 490 nm excitation and 520 nm emission wavelengths.

RNS production in cardiomyocytes was determined in an RNS indicator DAF-FM loading of the cells (5 μM; 60-min incubation at 37 °C). Loaded quiescent cells were examined with a laser scanning microscope (Leica TCS SP5, Germany). DAF-FM was excited at 488 nm and emission was collected at 520 nm. To maximize the level of NO, cells superfused with ZipNONO (100 μM) supplemented HEPES buffered solution.

All recordings with confocal microscopy were performed at 37 °C. To prevent photobleaching and cell damage, laser line was kept in 4–6% of maximal intensity.

### Determination of mitochondrial membrane potential

The mitochondrial membrane potential, MMP, was measured using a fluorescence-based method for tracing the behavior of mitochondria, as described previously [24]. Shortly, isolated cardiomyocytes were loaded with a membrane-permeant single wavelength fluorescence dye JC-1 (5 μM for 30 min) and imaged with a confocal fluorescence microscope (Leica TCS SP5). The probes were excited at 488 nm and the red fluorescence image was detected at both 535 nm and 585 nm and carbonylcyanide 4-(trifluoromethoxy)phenylhydrazone (FCCP; 5 μM) was used for calibration.

### Western blot analysis

All Western-blotting were performed in isolated cardiomyocytes. The cells were first harvested in lysis buffer, and then they were homogenated. The homogenates were centrifuged at 12,000×*g* for 15-min at 4 °C. The supernatant was used to determine the protein concentration by using the Bradford-assay. The equal amount of protein preparations were used on SDS–polyacrylamide gels, electrotransferred to polyvinylidene difluoride membranes, and blotted with a primary antibody against Mfn-1 (sc-166644, 1:1000), Mfn-2 (sc-100560, 1:1000), Fis-1 (sc-98900, 1:1000) and β-actin (Santa Cruz, sc-47778, 1:5000). Immunoreactive bands were detected by a chemiluminescent reaction (ECL kit, Amersham Pharmacia, USA).

### Determination of protein thiol oxidation level in isolated cardiomyocytes

In isolated cardiomyocytes, total and oxidized sulfhydryl (thiol) groups in proteins were measured with Ellman’s reagent, as described previously [20]. Briefly, for the measurement of total thiol-groups in cell homogenates, both for reaction mix and background reaction mix, cell lysate and 20% trichloroacetic acid mixtures were centrifuged for 10 min at 13,000 rpm. Both for total and oxidized thiol-group measurements, 2 mM 5,5′-dithiobis-(2-nitrobenzoic acid) were added to reaction mixtures and let them stand for 20 min. Then all reaction mixtures and background reaction mixtures were located into 96-well plates and absorbances were read using microplate-reader (SpectraMax Plus384) at 412 nm.

### Measurement of ADP/ATP ratio in isolated cardiomyocytes

The ADP to ATP ratio in isolated left ventricular cardiomyocytes was determined by using an ADP/ATP Ratio Assay Kit (ab65313) as described previously [18]. In here, the luciferase-catalyzed the conversion of ATP and luciferin to light, which subsequently measured using a luminometer. ADP level was measured by its conversion to ATP that was subsequently detected using the same reaction. Absorbances were read in SpectraMax Plus384 microplate reader at 570 nm.

### Assessment of aortic function

Isolated and cleaned thoracic aorta was sectioned into 3-mm long rings and then these samples were stretched to a 1-g initial tension and were equilibrated for 60 min in a bathing solution (Krebs–Henseleit solution as mM: NaCl, 119; KCl, 4.8; MgSO_4_, 1.2; CaCl_2_, 1.8; NaHCO_3_, 25; KH_2_PO_4_, glucose 10, bubbled with 95% O_2_ and 5% CO_2_). Tension development was measured by isometric force transducers (GRASS FT03) connected to an amplifier. Cumulative dose–response curves to phenylephrine hydrochloride (Phe; 10^−7^–10^−4^ M) for contractile activity and acetylcholine (Ach; 10^−7^–10^−4^ M) for relaxation activity were obtained.

### Chemicals and statistics

Chemicals are obtained from Sigma-Aldrich (St. Louis, MO) unless otherwise noted. The fluorescence probes were purchased from Molecular Probes, Eugene, OR. The results are expressed as means ± SEM. Statistical significance is evaluated by one-way ANOVA followed by Tukey post-test. The probability level of p < 0.05 is considered statistically significant.

## Results

### Systemic effects of DAPA administration in MetS rats

**Fig. 1:**
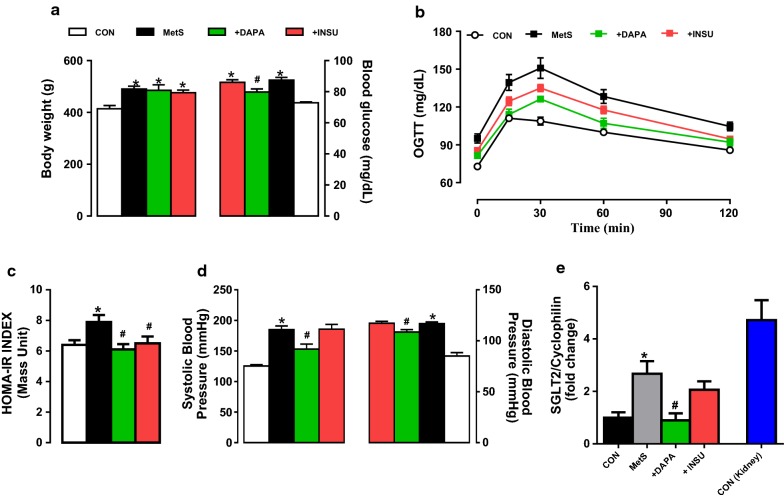
General effects of MetS rats treated with either DAPA or INSU. **a** Body weight (left) and blood glucose level of all groups. **b** Oral glucose tolerance tests (OGTT) by measuring blood glucose levels before and after orogastric gavage of 1 g/kg glucose administration at 15th min, 30th min, 60th min, 120th min to the rats. **c** The HOMO-IR index determined as described previously [16]. **d** The systolic (left) and diastolic (right) pressure changes measured from the tail. **e** Confirmation of DAPA beneficial effect in MetS rats directly targeting the heart by measuring mRNA levels of SGLT2 in tissue homogenates. Data presenting as mean (± SEM) values. The total number of rats/group; n = 15–17. Significance level at *p < 0.05 vs. CON group and ^#^p < 0.05 vs. MetS group

DAPA administration to MetS rats, compared with vehicle, modestly but significantly decreased morning-fed glucose values while INSU administration for the same period had no significant lowering effect on the blood glucose levels (Fig. 1a, right). However, both administrations could not induce any significant effect on body weight of MetS rats (Fig. 1a, left). Additionally, administration of DAPA, more prominently comparison with INSU did affect slightly but significantly (p < 0.05) the peak blood glucose levels measured at 15th, 30th, 60th, and 120th min during the OGTT monitoring as compared with vehicle-treated MetS rats (Fig. 1b). Both treatment also significantly preserved the increases in the HOMO-IR index of MetS rats during 2-week in vivo treatment (Fig. 1c).

**Fig. 2:**
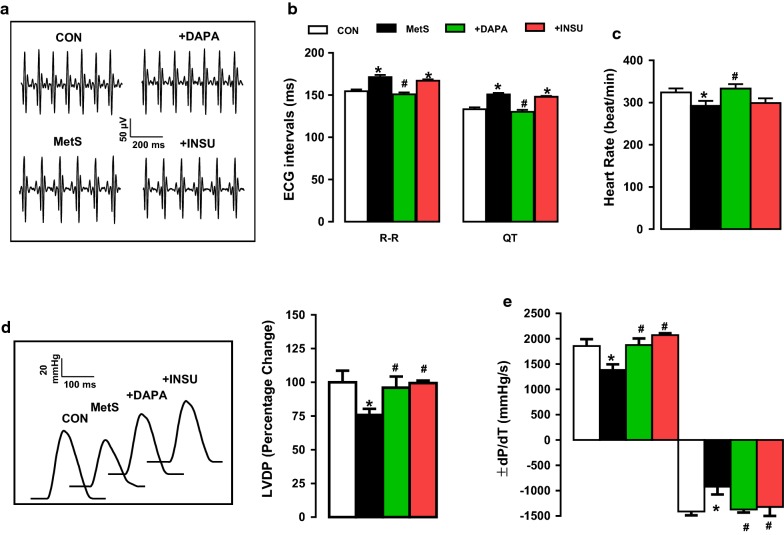
Assessment of in vivo electrical activity of the hearts from DAPA or INSU administrated Mets rats. **a** Representative in situ ECG traces and **b** calculated ECG parameters including the R–R and Q–T intervals from DAPA or INSU treated MetS group compared to either MetS or age-matched control rats. **c** Heart rate was estimated from ECG traces. The representative pressure changes (inset) of the LVDP changes (**d**) and the rates of pressure development and decay (± dP/dt) of LVDP (**e**). Data presenting as mean (± SEM) values. The total number of rats/group/protocol; n = 6–7. Significance level at *p < 0.05 vs. CON group and ^#^p < 0.05 vs. MetS group

Furthermore, treatment of MetS rats with DAPA but not INSU for 2-week induced marked decreases in both systolic and diastolic pressures compared with vehicle (Fig. 1d). Interestingly, although we have determined a marked shortening in QT-interval with increased heart rate in Mets rats with 22–24 weeks feeding [16], in here, we determined significantly prolonged RR- and QT-intervals in ECG recordings with modestly but significantly reduced heart rate in MetS rats with 28-week high carbohydrate feeding. These data can give as an indication of first adaptation period for high carbohydrate diet together with induction of insulin resistance, short QT-interval and high heart rate during relatively short feeding period (22–24 weeks; [25]), which may be followed with a chronic insulin resistance and remodelled heart function in the MetS rats with long QT-interval and low heart rate (Fig. 2b and c, respectively). DAPA but not INSU administration for 2-week, compared with vehicle, induced marked protection in ECG parameters of the MetS rats. The representative original ECG recordings are given in Fig. 2a.

To examine the presence of SGLT2 in rat heart, we performed mRNA measurements in heart tissue from MetS and control rats compared with their kidney tissue. As can be seen in Fig. 1e, the SGLT2 mRNA level in the heart from MetS rats was over the twofold increased comparison to controls. DAPA but not INSU treatment significantly normalized the increased mRNA level of SGLT2 in MetS rat heart. The detailed validation of SGLT2 in the rat heart tissue was given in Additional file 1: Figure S1.

### Effect of DAPA administration on hemodynamic parameters of heart in MetS rats

The hemodynamic parameters of heart such as left ventricular developed pressure, LVDP, changes (Fig. 2d) and the rates of pressure development and decay (± dP/dt) of LVDP (Fig. 2e) are determined in MetS groups comparison with the control (Con) group and the DAPA- or INSU-treated MetS groups comparison with the MetS group. The representative pressure changes are given as inset in Fig. 2d. As can be seen in these figures, the LVDP was significantly lower in MetS group (74 ± 5%) comparison to those of controls (100 ± 9%) while both + dP/dt and − dP/dt were slower in MetS rats (1384 ± 108 mmHg/s vs. − 920 ± 150 mmHg/s for + dP/dt vs. − dP/dt) than the controls (± 136 mmHg/s vs. − 1411 ± 75 mmHg/s for + dP/dt vs. − dP/dt). Either DAPA or INSU treatment of MetS rats rescued all these changes, fully (DAPA; 1876 ± 28 mmHg/s vs. − 1370 ± 60 mmHg/s for + dP/dt vs. − dP/dt and INSU; 2070 ± 34 mmHg/s vs. − 1320 ± 259 mmHg/s for + dP/dt vs. − dP/dt). Therefore, these hemodynamic data presented the cardioprotective effect of DAPA in insulin-resistant MetS rats, markedly, besides its beneficial effect on the electrical activity of the heart.

We performed experiments with aortic rings under phenylephrine, (Phe: 10^−7^–10^−4^ M) stimulation and acetylcholine (Ach: 10^−7^–10^−4^ M) relaxation. The contractile activity of aortic rings in MetS-group was not significantly different from that of control-group while no effect with DAPA-group (Additional file 2: Figure S2-A). We determined significantly depressed relaxation function of aortic rings in MetS-rats under Ach exposure (10^−7^–10^−4^ M) in a manner of cumulative application (about 75% vs. 100% in MetS-group vs. control group). DAPA administration also induced fully improvement in the depressed relaxation function of aortic rings observed in MetS rats (Additional file 2: Figure S2-B). However, the maximum response to highest Ach exposure (10^−4^ M) was about 70 ± 10% in the aortic rings from INSU treated MetS rats.

### Effects of DAPA administration on the electrical activities of cardiomyocytes

**Fig. 3:**
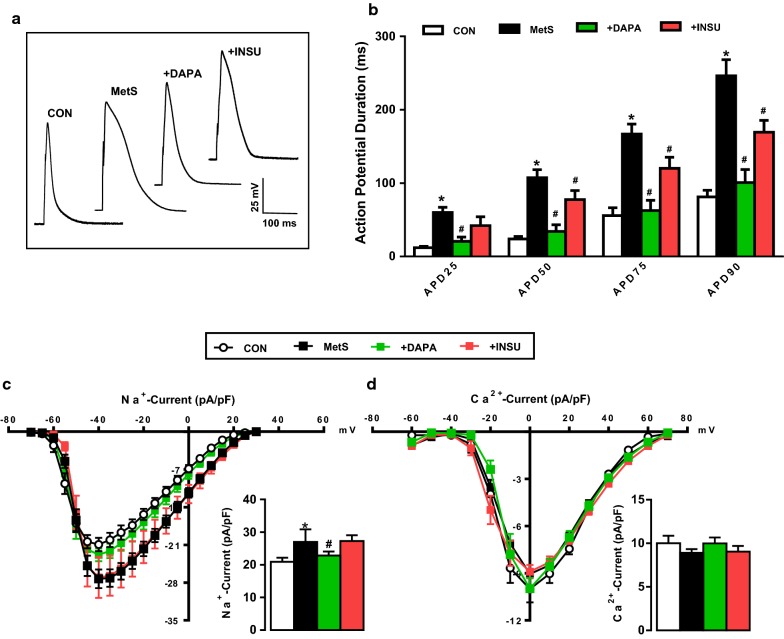
Effects of DAPA or INSU treatment on action potential parameters in freshly isolated cardiomyocytes. **a** Representative single cell action potential traces for all groups. **b** Action potential duration (APD) at 25, 50, 75, 90% (APD_25_, APD_50_, APD_75_, APD_90_) of repolarization are examined from DAPA or INSU treated MetS group compared to MetS and age-matched controls. The current–voltage relations (current–voltage, I–V, characteristics) of voltage-dependent Na^+^-channels (**c**) and L-type Ca^2+^**-**channels (**d**) for all groups. The calculated maximum current of Na^+^-channels (− 40 mV) and Ca^2+^-channels (0 mV) are given in the lower parts of the graphs as an inset. The total number of cells/group/protocol; n = 6–7. Significance level at *p < 0.05 vs. CON group and ^#^p < 0.05 vs. MetS group

Similar to changes in action potential parameters from streptozotocin-induced diabetic rat cardiomyocytes [26], we observed an important prolongation in action potential repolarization phases (at 25, 50, 75 and 90% of repolarization; AP_25_, AP_50_, AP_75_ and AP_90_, Fig. 3b) with no changes in the cell capacitance (260 ± 15 pF vs. 240 ± 9 pF for MetS vs. CON groups) and the resting membrane potential of cells (− 70 ± 1.1 mV vs. − 73 ± 0.9 mV for MetS vs. CON groups) with increased amplitude of APs (94.1 ± 2.0 mV vs. 83.3 ± 2.1 mV for MetS vs. CON groups) measured in > 40 cells using current-clamp mode of whole-cell patch-clamp technique. We observed significant improvements in the amplitude and the prolonged repolarizations of APs in either DAPA or INSU administered MetS rats compared with those of non-administered MetS rats. Of note, as also can be seen in Fig. 3b, the improvements at all repolarization times were more prominent in DAPA group comparison to INSU group. The representative original action potential recordings are given in Fig. 3a.

To determine the possible contribution of any change in the Na^+^-influx (via I_Na_) to the action potential amplitude, we determined first the I_Na_ in MetS rat cardiomyocytes. As can be seen in Fig. 3c, there was a slight but significant increase in the maximum value of this current measured at − 40 mV (Fig. 3c, inset) with no change in voltage-dependency, whereas DAPA but not INSU treatment restored this current in MetS group, significantly (Fig. 3c).

We also determined the L-type Ca^2+^-channel currents (I_CaL_) in cardiomyocytes from MetS rats to examine their possible contribution to action potential duration. As can be seen in Fig. 3d, there was no significant change in both the maximum value (lower part) and voltage-dependency (upper part) of this current measured at 0 mV (Fig. 3d) with no change in DAPA or INSU treated MetS cardiomyocytes.

**Fig. 4:**
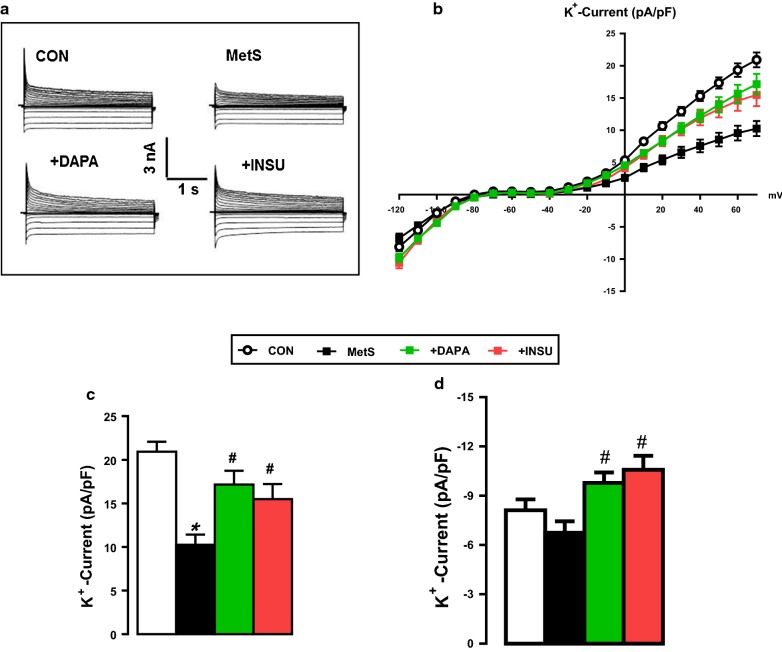
Effects of DAPA or INSU treatment on the voltage-dependent repolarizing K^+^-channel currents in freshly isolated cardiomyocytes. **a** Demonstration of representative original repolarizing K^+^-channel traces for all groups. The current–voltage relations (I–V characteristics) of K^+^ channels (**b**) and maximum outward (**c**, + 70 mV) and inward currents (**d**, − 120 mV) in DAPA or INSU treated MetS rats compared to MetS rats and age-matched control rats. The total number of cells/group/protocol; n = 12–15. Significance level at *p < 0.05 vs. CON group and ^#^p < 0.05 vs. MetS group

In another set of experiments, in order to clarify the possible contribution of the changes in voltage-dependent K^+^-channel currents (I_K_) to prolongations in AP repolarization phases, we first measured I_K_ in cardiomyocytes from MetS rats comparison with control group rats. As can be seen in Fig. 4a–d, the measured I_K_ in cardiomyocytes from MetS rats at between − 120 and + 70 mV voltage changes were found to be significantly depressed starting at positive voltages such as from 0 to + 70 mV (Fig. 4b). Both DAPA and INSU administration to MetS rats improved these currents, significantly (Fig. 4c). Interestingly, although the I_K_ at negative potentials did not change, these administrations induced a significant increase in I_K_ measured at − 120 mV (Fig. 4d). This effect of DAPA can be interpreted as its effect on the membrane potential to keep it at normal levels.

### DAPA or INSU administration affects the intracellular free Ca^2+^ changes in cardiomyocytes from MetS rats

**Fig. 5:**
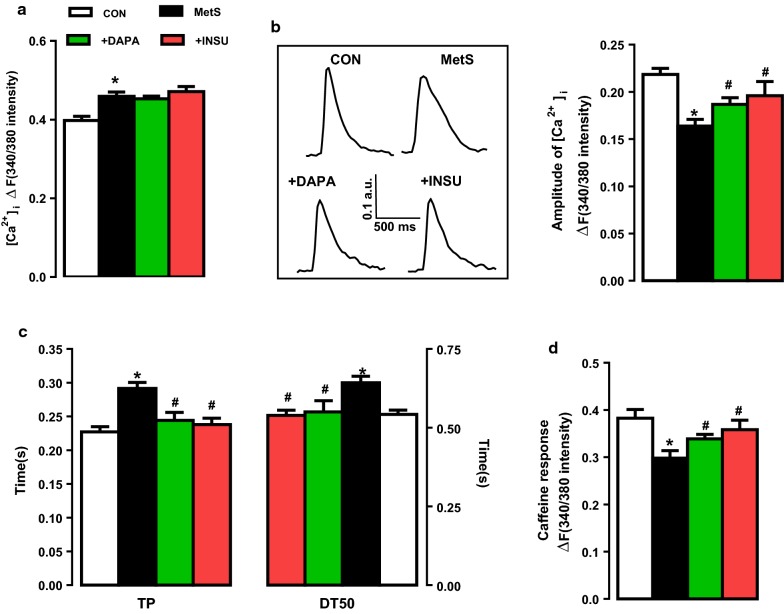
Changes in Ca^2+^ transients in DAPA or INSU treated MetS rats compared to MetS rats and age-matched controls rats in Fura-2AM loaded cardiomyocytes. The basal level of Ca^2+^ in resting cells (**a**), the representative original Ca^2+^ transients under electrical stimulation (**b**, left), the amplitude of transients Ca^2+^ changes and their corresponding kinetics under electrical-field stimulation (**b**, right), the time course of Ca^2+^-transients (**c**) in DAPA or INSU treated MetS rats compared to MetS rats and age-matched control rats. **d** Assessment of sarco(endo)plasmic reticulum (SR) Ca^2+^ content obtained following 10 mM caffeine applications. The total number of cells/group/protocol; n = 19–30. Significance level at *p < 0.05 vs. CON group and ^#^p < 0.05 vs. MetS group. *TP* time to peak, *DT50* decreasing time to 50% relaxation

We first determined the basal intracellular free Ca^2+^ level ([Ca^2+^]_i_) in Fura-2AM loaded cardiomyocytes as fluorescence intensity changes (as ΔF_340/380_). The average basal level of [Ca^2+^]_i_ was found to be significantly higher in MetS group comparison with the control group (Fig. 5a) while no effect with either DAPA or INSU treatment.

Figure 5b (inset) shows original recordings of global Ca^2+^ transients elicited in cardiomyocytes from four experimental group of rats. The averaged peak amplitude (as ΔF_340/380_) of Ca^2+^ transients was significantly smaller in MetS group than in the control group (Fig. 5b). The time to peak amplitude and the half-time for recovery of Ca^2+^ transients in MetS group were significantly longer than those of the controls (Fig. 5c). The responses to acute caffeine (10 mM) exposures were also significantly less in MetS group comparison with that of the control group (Fig. 5d). Both DAPA and INSU administration to MetS rats improved these values, significantly with the similar extends.

### Intracellular levels of free Na^+^ and H^+^ in isolated cardiomyocytes

**Fig. 6:**
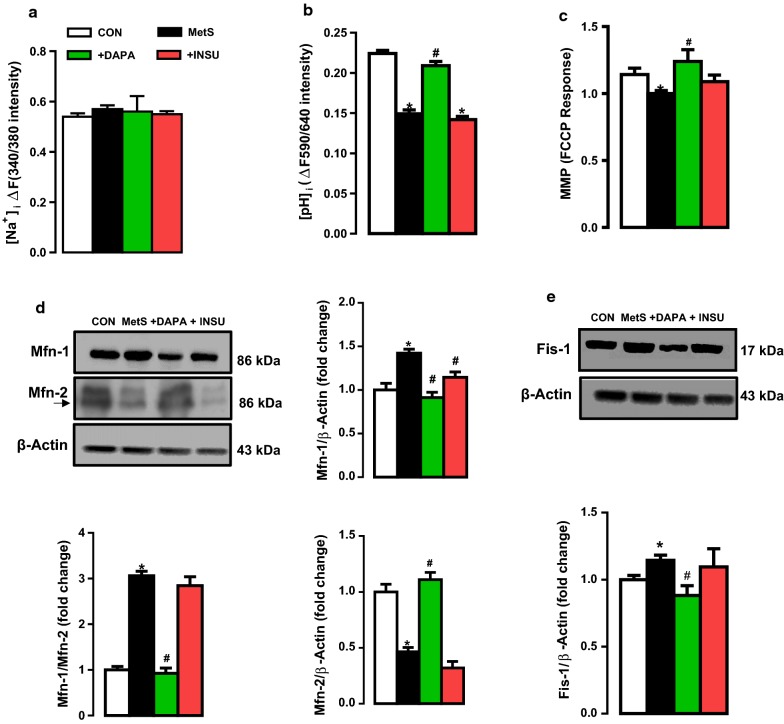
Confocal imaging of intracellular levels of free Na^+^ and H^+^ and analysis of mitochondrial function in DAPA or INSU treated MetS rats compared to MetS rats and age-matched controls. The level of free Na^+^ ([Na]_i_) estimated in cells loaded with Na^+^-sensitive SBFI florescent dye (**a**) and free H^+^ ([H^+^]_i_ or [pH]_i_) with SNARF-1AM (**b**). The mitochondrial membrane potential (MMP, as FCCP responses) in isolated JC-1 loaded cardiomyocytes (**c**). The expression levels of mitofusion proteins Mfn-1 and Mfn-2 and their ratio (**d**). The expression level of mitofission protein Fis-1 (**e**). The total number of cells/group/protocol; n = 10–19. Significance level at *p < 0.05 vs. CON group and ^#^p < 0.05 vs. MetS group

The averaged basal level of intracellular free Na^+^ ([Na^+^]_i_, measured as ΔF_340/380_ intensity change) was found to be similar in MetS group comparison with the control group (Fig. 6a). Neither DAPA nor INSU administration could affect the [Na^+^]_i_ in MetS rat cardiomyocytes.

In another set of experiments, we also determined the basal level of intracellular H^+^ (or intracellular pH, [pH]_i_ as ΔF_590/640_ intensity change) in cardiomyocytes from MetS rats comparison to those of control rats. As can be seen in Fig. 6b, the average [pH]_i_ was significantly less in MetS group comparison with the controls. DAPA but not INSU administration could improve this depressed [pH]_i_ to the control level.

### DAPA administration normalized the altered mitochondrial function in MetS rat cardiomyocytes

Since the previous studies demonstrated a role for mitochondrial redox signaling (showing via both ROS and RNS) in the etiology of MetS and highlighted a potential therapeutic agent to treat MetS in human subjects [27, 28], we determined the effect of DAPA treatment on mitochondrial membrane potential (MMP, as FCCP responses) in isolated JC-1 loaded cardiomyocytes from MetS rats. As can be seen in Fig. 6c, MMP was significantly depolarized in MetS group comparison with that of the control group. DAPA but not INSU treatment of MetS rats preserved the MMP level, significantly.

To further examine the role of DAPA on mitochondrial dysfunction, we also determined the expression levels of mitofusion proteins such as Mfn-1 and Mfn-2. As can be seen in Fig. 6d, the increased Mfn-1 and decreased Mfn-2 levels in MetS cardiomyocytes were normalized with DAPA treatment. Therefore, the threefold increased Mfn-1/Mfn-2 ratio is found to be fully normalized in cardiomyocytes from DAPA treated MetS rats.

In another set of experiments, we detected the expression level of mitofission protein Fis-1. As can be seen in Fig. 6e, the slightly but significantly increased level of Fis-1 was found to be normalized in DAPA treated Mets rat cardiomyocytes.

### DAPA administration normalized the increased cellular levels of both ROS and RNS in MetS rats

**Fig. 7:**
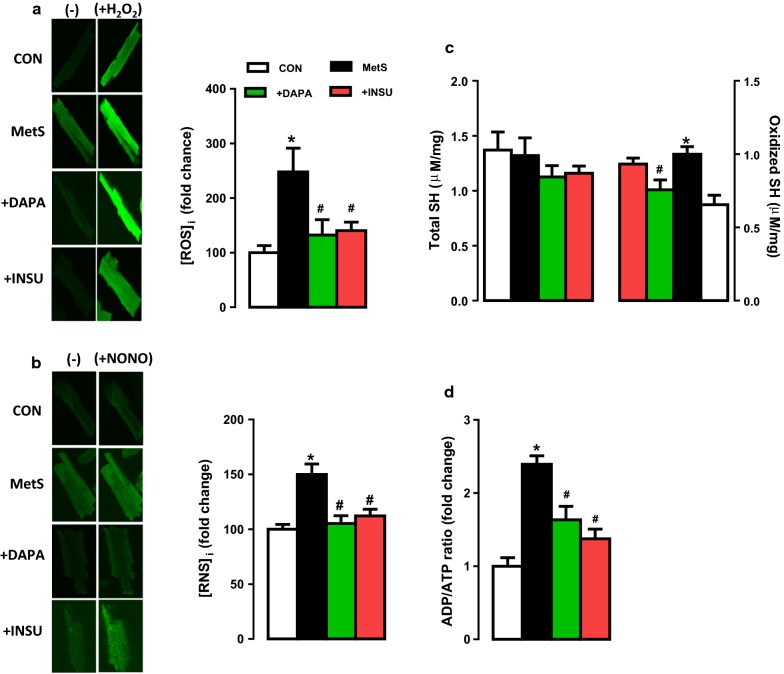
Assessment of oxidative stress status in cardiomyocytes in DAPA or INSU treated MetS rats compared to MetS rats and age-matched controls. Intracellular ROS ([ROS]_i_) (**a**) and RNS levels ([RNS]_i_) (**b**) imagined with confocal microscopy in cells loaded with either a specific dyes DCFDA or DAF, respectively. Maximal fluorescence intensity was achieved by a HEPES-buffered solution supplemented with H_2_O_2_ (100 μM) and NO donor ZipNONO, (100 μM). The free and total protein thiol levels (**c**) and the ADP/ATP ratio (**d**) in isolated cardiomyocytes. The total number of cells/group/protocol; n = 15–17. Significance level at *p < 0.05 vs. CON group and ^#^p < 0.05 vs. MetS group

In order to show a role of DAPA in mitochondrial redox signaling, we determined the cellular levels of ROS ([ROS_i_]) and RNS ([RNS_i_]) in cardiomyocytes loaded with specific fluorescence probes for ROS and RNS production as fluorescence intensity changes. ROS production was about 2.5 fold high in MetS rats comparison with the controls (Fig. 7a). Either DAPA or INSU treatment of MetS rats normalized these values to those of controls. Similarly, the RNS production in cardiomyocytes from MetS rats was about 1.5 fold high compared to those of controls, while these two treatments of MetS rats induced almost a full normalization in these values (Fig. 7b). The representative fluorescence intensities for ROS and RNS are given on the left sides of the bar-graphs.

### DAPA treatment prevented the protein thiol oxidation and increase in ADP/ATP ratio in MetS rat cardiomyocytes

In the last group examinations, we first determined the levels of protein thiol (SH) oxidation levels in MetS rat cardiomyocytes by measuring the total and oxidized SH-groups. The oxidized SH-groups were significantly high in MetS group compared to those of controls while DAPA but not INSU treatment could prevent these oxidized SH-groups, significantly with no change in the total SH-groups in these groups (Fig. 7c).

We also determined the level of ADP/ATP ratio in MetS rat cardiomyocytes. As can be seen in Fig. 7d, this ratio was found to be 2.5 fold high in MetS group compared to those of controls. Either DAPA or INSU treatment of MetS rats preserved this ratio, significantly.

## Discussion

In this study, our data suggested an important information about the molecular mechanism of an SGLT2-inhibitor DAPA-treatment benefits on cardiovascular function in MetS rats via targeting directly heart, behind its glucose-lowering effect. Our findings also suggest a new insight into DAPA-associated cardioprotection in MetS rats including suppression of prolonged ventricular-repolarization through augmentation of mitochondrial function and oxidative stress. Comparison to INSU-treatment, the DAPA-treatment induced a significant augmentation in the low heart-rate together with a depressed left ventricular function and relaxation of the aorta. Our finding related to the general effects of SGLT2 inhibitors are in line with previously published data. In this regard, Lahnwong et al. [29] recently reviewed extensively the potential mechanisms responsible for the cardioprotective effect of SGLT2 inhibitors, including DAPA, taken into consideration in both human and animal studies. Similar to our findings, the data generated from most of studies in this field demonstrated the benefits of these inhibitors on fasting blood glucose level, HOMA-IR as well as systolic and diastolic blood pressure [30–33]. However, Kusaka et al. have shown that another SGLT2 inhibitor Empagliflozin lessened cardiac injury and reduced visceral adipocyte hypertrophy as well as ameliorated cardiac hypertrophy and fibrosis, in association with the attenuation of cardiac oxidative stress and inflammation in prediabetic rats with MetS without significant changes in blood pressure, heart rate or sympathetic activity [34]. Moreover, Joubert et al. [35] have emphasized that DAPA prevents cardiomyopathy in the hypertrophic heart through the glucose-lowering action and control of glucose overload of DAPA. Our data are also in line with previously published clinical findings. The important clinical trials demonstrated the important benefits of SGLT2 inhibitors, without any side effect, including lower rate of the primary composite cardiovascular outcome [10, 36] as well as achievement of superior diabetes-control and blood pressure, at least, related with the early stages of vascular remodelling [32].

### SGLT2 inhibitor DAPA exhibited marked benefits on cardiovascular function in MetS rats through its effect on cardiac ionic homeostasis

We, here for the first time, reported that DAPA-treatment provided marked cardioprotection by improving prolonged QT-interval in ECG and depressed LVDP in MetS rats through, at least, augmentation in ionic-mechanisms at the cellular level. DAPA-treatment-associated improvements in prolonged ventricular repolarization seemed to be through the augmentation in depressed voltage-gated K^+^-channel currents (I_K_). We also observed significantly increased Na^+^-influx via not only voltage-gated Na^+^-channels but also increased the expression level of SGLT2 in cardiomyocytes from MetS rats. Interestingly, although the amplitude of action potentials was significantly high in MetS cardiomyocytes being parallel to increased Na^+^-influx, the basal level of intracellular free Na^+^ ([Na^+^]_i_) was not significantly different in these cardiomyocytes. However, Lambert et al. [33] demonstrated elevated [Na^+^]_i_ in both diabetic humans and rat model, providing a hypothesis via an enhanced SGLT in these samples. Our observation of similar [Na^+^]_i_ in both MetS and control rats may arise due to several different but crossing mechanisms. First, we have observed a significant decrease in the level of intracellular free H^+^ ([H^+^]_i_ or pH_i_) with a significant increase in intracellular free Ca^2+^ ([Ca^2+^]_i_) in MetS cardiomyocytes. These observations are supported by previously published ones. It seems that the increased [Ca^2+^]_i_ which occurs due to not Ca^2+^-influx into cell by voltage-dependent Ca^2+^-channels, but most probably via the reverse-mode action of the Na^+^/Ca^2+^ exchanger (NCX) may balance the level of [Na^+^]_i_ in MetS cardiomyocytes. Another explanation can be due to the increased activity of Na^+^/H^+^ exchanger (NHE) in MetS rat heart [37]. Indeed, in support of these two possible hypotheses, we detected both an increase in basal [Ca^2+^]_i_ and decrease in basal [H^+^]_i_ in MetS rat cardiomyocytes. SGLT2 inhibition markedly normalized and/or protected these changes in MetS rat heart. Of note, although we did not determine the other parameters, contributing to the intracellular ionic homeostasis, others have already documented an important role of SGLT2 inhibitors on Ca^2+^-handling proteins. SGLT2 inhibitors could provide positive effects on depressed LV in hyperglycemic samples via affecting activities of several proteins responsible from Ca^2+^-homeostasis, such as SERCA2a, CaMKII, phosphorylation level of RyR2. Therefore, it significantly could reduce SR Ca^2+^-leak and improve the depressed contractility [31, 38]. In these regards, our present data are nicely associated with previously published ones, because we have reported here that any reduction of myocardial [Na^+^]_i_ by inhibition of NCX or NHE improves the heart dysfunction [39]. More importantly, a recent study identified an important role of SGLT2 inhibitor through its direct cardiac effects by lowering myocardial [Na^+^]_i_ and [Ca^2+^]_i_ and enhancing mitochondrial [Ca^2+^], which occurs in an impairment of myocardial NHE flux, independent of SGLT2 activity [39]. Although we did not check these last parameters, we observed a significant recovery in a mitochondrial function in DAPA-treated MetS rat cardiomyocytes. Therefore, all together, SGLT2 inhibitor DAPA may inhibit a myocardial NHE leading to increased mitochondrial [Ca^2+^] and decreased cytoplasmic [Ca^2+^]_i_ and [H^+^]_i_ through mitochondrial NCX activity in MetS rat heart. In line with these findings above, Uthman and co-workers recently demonstrated a direct effect of DAPA on the heart via inhibition of NHE flux and followed by reducing [Na^+^]_i_, which came up a conclusion on its potential side to combat heart failure and diabetic cardiac dysfunction [40].

### Suppression of increased cardiac oxidative stress with DAPA contributes to its cardioprotection action in MetS rats

This present study showed that treatment with DAPA led to improvement in indices of deleterious cardiac function in an insulin resistant metabolic syndrome rat model with slight but significant hyperglycemia. Our findings also suggested that DAPA-treatment of MetS rats indicated a significant protection against high amount of ROS and RNS productions in isolated cardiomyocytes. These protections could provide a further protection against the oxidation of protein thiols. In line with this, we detected normalized and/or oxidized thiol levels in DAPA-treated MetS rat cardiomyocytes. In support of these findings, some studies have reported that SGLT2 inhibitors, including DAPA, might act as antioxidants by decrease in a cardiac oxidative stress, out of glucose lowering effects [34, 41, 42].

Additionally, it has been well documented that the prolonged cardiomyocyte action potential in diabetic mammalians is due to depressed I_K_, at most due to increased oxidative stress, at both systemic and cellular levels [26, 43–48]. Indeed, this current study is nicely in line with those previous findings. Furthermore, In this regards, recently published studies have already reported the antioxidant-like action of SGLT2 inhibitors [41]. In the study of Lee et al., myocardial ischemic rats were fed with DAPA for 2 days and a significant attenuation in ROS and RNS levels has been came up in cardiac tissues. Those evidences, including our current data with DAPA support the potential role of DAPA in MetS-associated heart dysfunction as antioxidant and inflammatory modulators through suppression of induction of oxidative stress, independent of its SGLT2 and glucose lowering effects.

### Improvement of mitochondrial function with DAPA contributes to the cardioprotective action of SGLT2 inhibitors in MetS rats

Since mitochondria is important to maintain a physiological cardiac function via its roles in ROS production and Ca^2+^-homeostasis due to their roles, in Ca^2+^ homeostasis, and in the production of energy and ROS [49]. On the other hand, the impaired mitochondrial function and dynamics are observed in diabetic patients and leads to myocardial contractile dysfunction [50]. In this present study, these parameters were found to be altered in our heart samples, and therefore, we focused on how DAPA-treatment could affect the mitochondrial dysfunction. We observed a normalization in mitochondrial membrane potential and well-controlled Ca^2+^-homeostasis, less ROS and RNS production and normalized ADP/ATP ratio as well as normalized fusion–fission proteins, such as normal protein expression levels of Mfn-1, Mfn-2 and Fis-1 with DAPA-treatment. Therefore, we may propose that mitochondria is one of the targeted subcellular organels in cardiomyocytes affected by insulin resistance and they could respond to the treatment of SGLT2 inhibitors [42, 51]. In this regard, our present proposal is in line with previously published data suggesting a mitochondrial dysfunction and the pathological progression of diabetic cardiomyopathy [52]. Indeed, impaired mitochondrial function and dynamics were observed in diabetic patients and they lead to myocardial contractile dysfunction. Of note, the previous studies have already documented that Mfn-2 links mitochondrial and SR function with insulin signaling and is essential for normal glucose homeostasis [15]. Furthermore, it has also been also suggested that the levels of human Fis-1 at the mitochondrial outer membrane could responsible in the regulation of mitochondrial morphology [53].

All together with already known documents in literature, these results highlighted an important benefical effects of DAPA-treatment on mitochondrial function, independent of its systematic actions. In the support of our study, the recent some elegant experimental studies have reported their direct effects on cardiomyocytes, through their preserving action on mitochondrial function, although the most of cardioprotective effects of SGLT2 inhibitors are mostly suggested to be systemic effects via their hemodynamic and metabolic actions [42, 51, 54]. Particularly, the study of Habibi et al. have demonstrated that a 4-week treatment with DAPA attenuated the increase in cardiac mitochondrial fission parallel to the decrease in mitochondrial fusion as evidenced by altered protein expressions such as Mfn-2 and Fis-1 in obese-insulin resistant rats undergoing cardiac ischemia–reperfusion injury [51]. These previous observations were also confirmed in this present study in DAPA-treated MetS rats, as well.

## Limitations

In this present current study by uses of MetS mimicking rats, we demonstrated that DAPA-treatment of these rats comparison to those of INSU-treatment significantly augmented all systemic parameters, including electrical and mechanical activities of the heart. Furthermore, we provided an important evidence related with DAPA effects at cellular level, including ionic currents associated with cardiomyocyte electrical activity, Ca^2+^-homeostasis, increased oxidative stress and mitochondrial function. Although our present data can provide new insights about the SGLT2 inhibitor cardioprotective effects in animal models, there are still some limitations in our study need to acknowledge. First, the numbers of animals used i here were relatively small and the duration of treatment was limited to 2 weeks while one DAPA dose was used for the treatment. These facts might explain why we observed no effect on body weight and there was small effect on blood glucose and OGTT with DAPA treatment. Similarly, we did not detect any positive effect with INSU-treatment in these MetS-rats. Yet, these reasons can be due to short and limited time-period as well as not enough INSU dose for these MetS-rats. It seems, longer periods of treatments and/or different doses may require for a fully investigation of their exact actions in these animal models. Indeed, in vivo physiological settings are more complex than the real living body. Therefore, the clinical relevance of our findings deserves further investigation. It would be interesting to evaluate the potential effects of this drug in patients with early-stage insulin resistant overweight humans. Nevertheless, the findings of this study in animal models align well with recent clinical trial outcomes and further suggest a potential clinical utility for DAPA in the treatment of cardiac dysfunction in any type of pathological condition.

## Conclusions

This present study shows that treatment with DAPA led to improvements in the depressed left ventricular function, and prolonged Q–R interval and low heart-rate in a MetS-rat model with slight but significant hyperglycemia and severe insulin resistance. Our study has reported that SGLT2s exist in the heart, functioning independent of those of others in mammalians. Furthermore, our findings with DAPA, more prominently than INSU, on ionic mechanisms, oxidative stress status and mitochondrial function provide an important information about its a direct cardioprotective action. Collectively, the results of this investigation support the hypothesis that treatment with the SGLT2 inhibitor, DAPA, improves cardiac dysfunction function in the setting of MetS, diabetes, or obesity even in the little effect on high blood glucose and insulin resistance. Altogether, this study provides a new insight into DAPA-associated cardioprotection in MetS-rats through augmentation of mitochondrial function and oxidative stress via improvement of fusion–fission proteins, behind its glucose-lowering effect.

## Additional files

**Additional file 1.** Validation of SGLT2 in the rat heart tissue. (A) Primer pairs used for quantitative real-time PCR to assess steady-state mRNA level of SGLT2 (208 kb) both in heart and kidney tissues. (B) Analysis of DNA expression of SGTL2 in heart and kidney tissues by agarose gel electrophoresis.

**Additional file 2.** Effects of DAPA treatment on contractile activity of aortic rings. (A) Contractile responses of aortic rings to phenylephrine, Phe (10^−7^–10^−4^ M) stimulation in a manner of cumulative concentration applications with EC_50_ values. (B) Aortic rings, following pre-contracted with 100 µM Phe, are relaxed with acetylcholine, Ach (10^−7^–10^−4^ M) as a manner of cumulative concentration. The EC_50_ values from DAPA treated MetS comparison with those of MetS rats or control rats are given in tables as an inset. The maximum responses to Ach stimulation with high concentrations in MetS are markedly less compared to those of controls, while DAPA treatment of this group induced significant preservation of these depressed responses. The total number of rats for aortic rings/group; n=5-7. Significance level at *p<0.05 *vs.* CON group or MetS group.

## Abbreviations

CaMKII: Ca^2+^/calmodulin-dependent kinase II
DAF-FM: 4-amino-5-methylamino-2,7-difluorofluorescein
DAPA: dapagliflozin treatment
± dP/dt: rates of pressure development and decay
ECG: electrocardiography
FCCP: carbonylcyanide 4-(trifluoromethoxy)phenylhydrazone
Fis-1: mitochondrial fission-1
INSU: insulin treatment
LVDP: left ventricular developed pressure
MetS: metabolic syndrome
Mfn-1: mitofusin-1
Mfn-2: mitofusin-2
MMP: mitochondrial membrane potential
NCX: Na^+^/Ca^2+^ exchanger
NHE: Na^+^/H^+^ exchanger
RNS: reactive nitrogen species
ROS: reactive oxygen species
RyR2: cardiac ryanodine receptors
SBFI: sodium-binding benzofuran isophthalate, an AM-ester
SERCA2a: cardiac sarcoplasmic reticulum Ca^2+^ ATPase
SGLT2: sodium-glucose cotransporter-2
SNARF-1: carboxylic acid, acetate, succinimidyl ester
SR: sarcoplasmic reticulum
T2DM: type 2 diabetes
[Na^+^]_i_: the basal level of intracellular free Na^+^
[Ca^2+^]_i_: the basal level of intracellular free Ca^2+^
[H^+^]_i_: the basal level of intracellular free H^+^
