# Supplementary figures and images for: A SGLT2 inhibitor dapagliflozin suppresses prolonged ventricular-repolarization through augmentation of mitochondrial function in insulin-resistant metabolic syndrome rats
Source: Cardiovasc Diabetol. 2018 Nov 17;17:144. doi: 10.1186/s12933-018-0790-0 (PMC6240275; doi:10.1186/s12933-018-0790-0)

## Slide 1
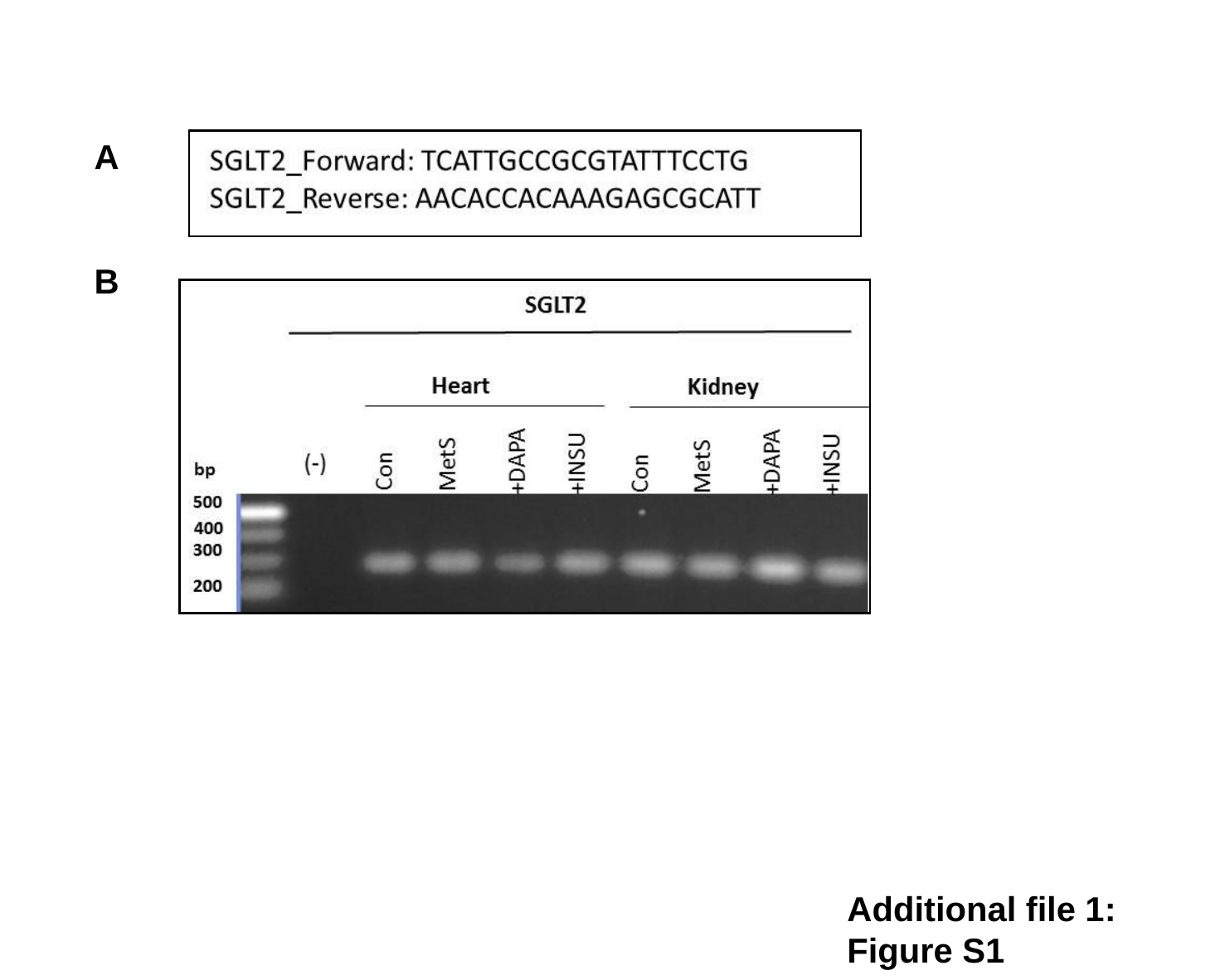

A
B
Additional file 1: Figure S1

Supplement: Supplementary file 1 — Additional file 1. Validation of SGLT2 in the rat heart tissue. (A) Primer pairs used for quantitative real-time PCR to assess steady-state mRNA level of SGLT2 (208 kb) both in heart and kidney tissues. (B) Analysis of DNA expression of SGTL2 in heart and kidney tissues by agarose gel electrophoresis. [file 12933_2018_790_MOESM1_ESM.pptx]

## Slide 1
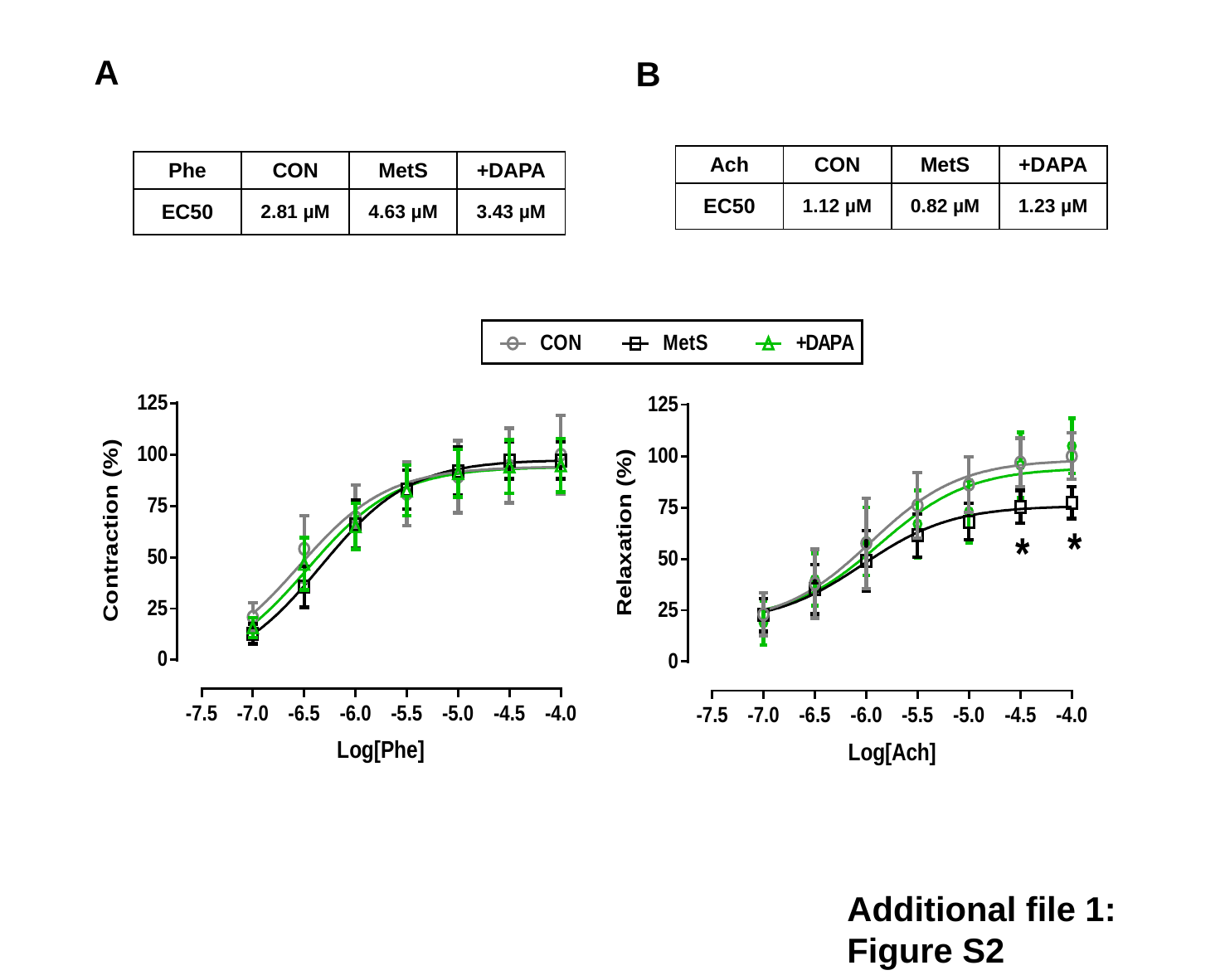

A
B
| Ach | CON | MetS | +DAPA |
| --- | --- | --- | --- |
| EC50 | 1.12 µM | 0.82 µM | 1.23 µM |
| Phe | CON | MetS | +DAPA |
| --- | --- | --- | --- |
| EC50 | 2.81 µM | 4.63 µM | 3.43 µM |
Additional file 1: Figure S2

Supplement: Supplementary file 2 — Additional file 2. Effects of DAPA treatment on contractile activity of aortic rings. (A) Contractile responses of aortic rings to phenylephrine, Phe (10−7–10−4 M) stimulation in a manner of cumulative concentration applications with EC50 values. (B) Aortic rings, following pre-contracted with 100 µM Phe, are relaxed with acetylcholine, Ach (10−7–10−4 M) as a manner of cumulative concentration. The EC50 values from DAPA treated MetS comparison with those of MetS rats or control rats are given in tables as an inset. The maximum responses to Ach stimulation with high concentrations in MetS are markedly less compared to those of controls, while DAPA treatment of this group induced significant preservation of these depressed responses. The total number of rats for aortic rings/group; n=5-7. Significance level at *p<0.05 vs. CON group or MetS group. [file 12933_2018_790_MOESM2_ESM.pptx]
